# The Low-Temperature Sol-Gel Synthesis of Metal-Oxide Films on Polymer Substrates and the Determination of Their Optical and Dielectric Properties

**DOI:** 10.3390/nano12234333

**Published:** 2022-12-06

**Authors:** Lyudmila Borilo, Vladimir Kozik, Alexander Vorozhtsov, Viktor Klimenko, Olga Khalipova, Alexander Agafonov, Tatiana Kusova, Anton Kraev, Yana Dubkova

**Affiliations:** 1 National Research Tomsk State University, 36 Lenin Avenue, 634050 Tomsk, Russia; 2 Krestov Institute of Solution Chemistry of the Russian Academy of Sciences, 1 Akademicheskaya Street, 153045б Ivanovo, Russia; 3 Ivanovo State University of Chemistry and Technology, 7 Shremetevsky Ave, 153000 Ivanovo, Russia

**Keywords:** thin metal-oxide films, heterostructures, transparent electroconductive coatings, silver nanowires, titanium dioxide

## Abstract

Photoactive, optically transparent heterostructures from silver nanowires and titanium dioxide were obtained by the sol-gel method on the surface of a polyethylene terephthalate film. The characteristics of optical transmission on the wavelength and those of dielectric permittivity, conductivity and dissipation on frequency in the range of 25–1,000,000 Hz were investigated.

## 1. Introduction

Transparent conductive electrodes (TCFs) are used in various optoelectronic devices such as OLED, solar batteries and touch screens as they determine characteristics of these devices such as light transmission, absorption, injection and emission [1,2,3]. A promising research direction now is creating flexible optoelectronic devices on transparent polymer substrates as, in addition to the convenience of application of such devices, another advantage of their use consists in replacing conventional vacuum technologies of production with alternative methods, such as 2d printing and the deposition of transparent conductive layers by solution technologies. Among the widely used electroconductive transparent oxides, indium tin oxide (ITO), most often applied in TCF, has low resistance and a high transmission coefficient. However, bending an ITO film electrode leads to its damage, and it loses losing electric characteristics. Moreover, ITO electrodes are produced on expensive vacuum equipment, increasing the prices of the goods in which this material is used. Among other materials that are considered to be potential alternatives to ITO are: silver nanowires, carbon nanotubes and graphene and metal grids [4,5,6,7]. These materials make the electrode flexible, whereas nonflexibility is among the ITO electrodes’ drawbacks. However, layers of graphene or carbon nanotubes as well as metal grids deposited on the surface of flexible transparent substrates have low electric and optical properties compared with ITO and high costs of production and dispersion into homogeneous suspensions without bundles or aggregates (carbon nanotubes) or separate sheets (graphene) and thus remain at the research stage [8,9]. One of the advantages of silver nanowires over the other above-mentioned alternative materials is that TCFs can be produced by employing a very simple coating process, which makes their production costs low, while the nanowire coating resistance and transmission characteristics are similar to those of conventional ITO electrodes. In particular, silver nanowire TCFs are suitable for producing flexible TCFs because their resistance changes under mechanical deformation and the layers of silver nanowires can be formed on TCF at low temperatures [10].

We have earlier studied the optical and dielectric properties of nanocomposites obtained on the surface of a transparent polyethylene terephthalate film by the layer-by-layer method combining the sol-gel technology and colloid-chemical reduction of silver and gold ions and alternating them with layers of titanium dioxide films. We have obtained optico-dielectric characteristics of coatings from titanium dioxide and TiO_2_, TiO_2_/Ag and TiO_2_/Ag/TiO_2_ silver anisotropic nanoparticles and found the interconnection of the optical transmission with the frequency dependence of the dissipation factor for these materials [11,12].

The aim of this study is to identify regularities of mutual effects of spectral and dielectric characteristics of thin metal-oxide films on the spectral and dielectric properties of heterostructures obtained by combining films with different conductivity types and optical characteristics. We also aim to develop a low-temperature method of obtaining photoactive metal-oxide semiconductor films on flexible transparent polymer substrates.

One of the goals of the study was to obtain transparent electroconductive coatings by using nanoheterostructures of silver nanowires and titanium dioxide films on a flexible transparent substrate from polyethylene terephthalate. Another task was to compare the effect of combining isotropic and anisotropic silver fillers of films on their optical and dielectric properties.

Metal nanowires of the same size can be synthesized by different chemical methods, among which the most astonishing results are achieved by the polyol process [13,14]. In the polyol synthesis, silver ions are reduced as a result of the following reactions in heated ethylene glycol:

2HOCH_2_CH_2_OH → 2CH_3_CHO + 2H_2_O
2Ag^+^ + 2CH_3_CHO → CH_3_CHO-OHCCH_3_ + 2Ag + 2H^+^

**Figure 1:**
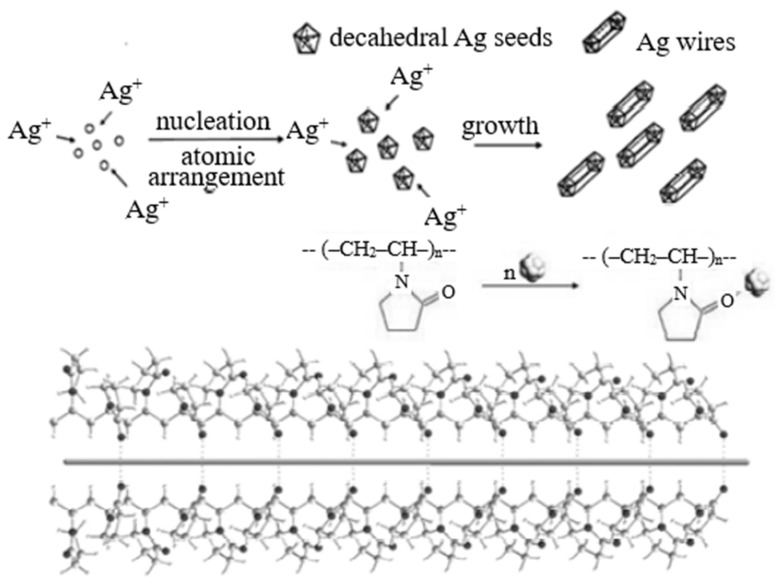
The process of silver nanowire formation in polyol synthesis.

It is assumed that the process of silver nanowire formation in polyol synthesis follows the scheme shown in Figure 1 [15,16].

The authors studying silver nanostructures synthesized by the polyol method have shown that the process of initial nucleation has a great effect on the final product morphology [13]. The process of crystallization nuclei formation is normally controlled by adding inorganic ions or organic molecules to the reaction system [17]. The authors of works [18,19] synthesized different silver nanostructures including nanocubes, nanowires and nanotetrahedrons using the polyol process with a chloride addition known as “oxidative etching of nuclei”. By changing the concentration and synthesis duration it is possible to control the diameter of silver nanowires [13]. At the same time, changes in the concentration have a greater effect on product morphology, whereas the possibilities to control the diameter by changing the reaction duration are quite limited [13,14,18,19,20,21,22,23,24].

## 2. Materials and Methods

### 2.1. Synthesis of Components for Consecutive Deposition of Electroconductive Photoactive Layers

The TiO_2_ sol was prepared by the low-temperature sol-gel method using titanium tetraisopropylateTi(OC_3_H_7_)_4_ (98%, Aldrich, St Louis, MO, USA) as a precursor [25]. To do so, 10 mL titanium isopropylate was slowly added, by drops and under constant mixing, to bid stilled water (100 mL) heated to 80 °C. This immediately produced a white fluffy precipitate. The precipitate suspension was mixed with nitric acid (65% HNO_3_, Aldrich, St Louis, MO, USA) at the ratio [Ti]/[H^+^] = 2.5. The obtained colloid was mixed at 70 °C for 4 h with the aggregated amorphous precipitate, due to peptization and heat effects, transformed into a transparent colloidal opalescent TiO_2_ sol that was used for forming films on the surface of the transparent polyethylene terephthalate substrate and obtaining a dry powder.

**Figure 2:**
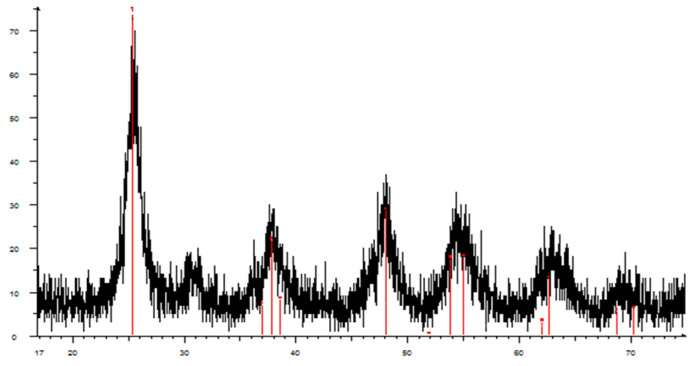
X-ray diffraction analysis of titanium dioxide TiO_2_ powder obtained by low-temperature sol-gel.

Figure 2 presents the XRD (D8 Advance, Bruker, Germany) pattern of prepared sample. The qualitative phase composition was determined using the PCPDFWIN database, the full-profile analysis program POWDER CELL 2.4. and the data from the crystallographic and crystallochemical database for minerals and their structural analogs (URL http://database.iem.ac.ru/mincryst (accessed on 1 October 2022)).

**Figure 3:**
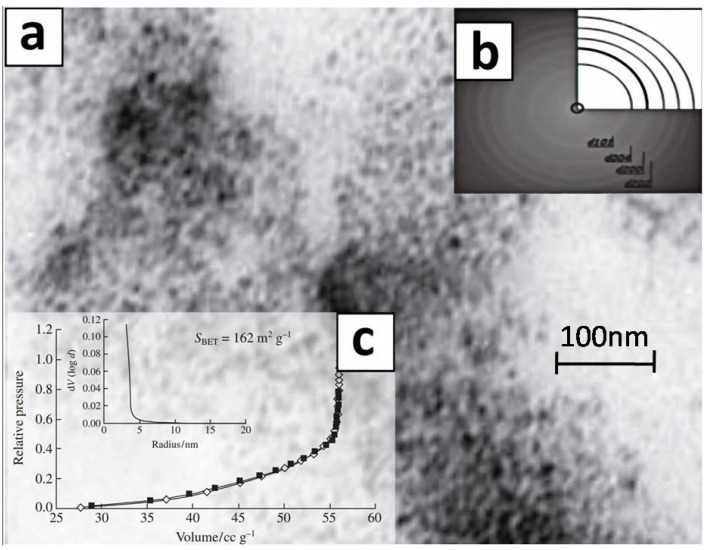
Characteristics of titanium dioxide nanoparticles obtained by low-temperature sol-gel method: (**a**) TEM-image; (**b**) general diffraction; (**c**) nitrogen sorption isotherm and pore size distribution.

The TEM image (TEM JSM-6510LV, JEOL Ltd, Japan) of titanium dioxide nanoparticles are shown in Figure 3. The sample has a homogeneous structure with a narrow particle size distribution (BET method, NOVA 1200e gas sorption analyzer, Quantachrome, Boynton Beach, FL, USA).

**Figure 4:**
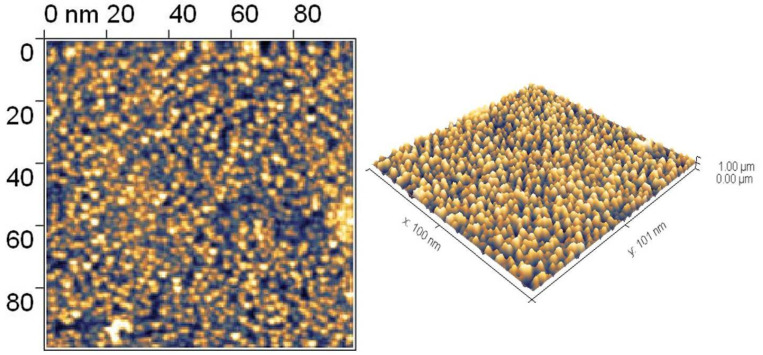
2D and 3D AFM-image of film surface.

The TiO_2_ film morphology was analyzed by atomic-force microscopy (SOLVER P47H-PRO, NT-MDT, Russia) (Figure 4). The obtained data indicate the uneven distribution of titanium dioxide nanoparticles in the film, which enables the effective interphase interaction of titanium dioxide with the silver nanoparticles. Additionally, the narrow size distribution of titanium dioxide nanoparticles with the maximum around 16 nm contributes to the formation of tight contacts on the interphase boundary TiO_2_-Ag.

### 2.2. Development and Optimization of Conditions of Polyol Synthesis of Silver Nanowires as Fillers of TiO_2_/Ag and TiO_2_/Ag/TiO_2_ Nanoheterostructures

Our polyol synthesis technique included the following original reagents: ethyleneglycol (EG) (Aldrich, St. Louis, MO, USA) as the solvent and reducing agent, AgNO_3_ (Aldrich, St. Louis, MO, USA) and polyvinylpyrrolidon- PVP (MW = 55,000) (Aldrich, St. Louis, MO, USA). KCl (Aldrich, St. Louis, MO, USA) and K_4_Fe(CN)_6_ were used as intensifiers of the anisotropic growth of Ag nanowires during the synthesis procedure.

The syntheses conducted in advance, in which the quality of the obtained nanowires was controlled by optical microscopy, showed that the optimal ratio of the components ensuring the largest yield of the target product with the minimum content of Ag isotropic nanoparticle impurities during the synthesis was: In a typical procedure of synthesizing silver nanowires (Ag_NW), 1.3 g of PVP was added to 6.67 mL of EG and heated over an oil bath (at the temperature of 150 °C) for 1 h with a magnetic agitator (260 rpm). Then, 13.3 mL of a solution containing 1 g AgNO_3_ and 0.00213 g KCl in EG precooled in a refrigerator was added to the mixture by drops from a light-proof syringe. The solution color changed during the synthesis procedure from transparent colorless to yellow (over 1 min), then it became orange-red (over 3 min), then gradually turned green (over 5 min) followed by dimming and gradual transition from green to brown-red (over 30 min) and, finally, to a nontransparent grey-crème color. After Ag nanowires were formed, the reaction was stopped by cooling the reaction vessel in a water bath. The centrifuged sediment was washed with acetone and water to remove the excess EG and PVP, dried and redispersed in isopropyl alcohol.

Silver nanowires were also obtained via microwave treatment of the systems used earlier in the synthesis with direct heating. In this case, solutions of silver nitrate, PVP, KCl or K_4_Fe(CN)_6_ in ethylene glycol were mixed util complete dissolution was achieved in a dry box in a nitrogen atmosphere (Ag_NW_MW). Then they were poured into a Teflon vessel with a lid. This reactor was placed into a microwave oven (sample preparation system MC-6 (OOO NTF Volta, St. Petersburg, Russia) at atmospheric pressure) with power regulation from 200 to 1000 W. To prevent excessive pressure rise inside the reactor, the microwave during the synthesis was heated with power pulses for 1.0 min with subsequent relaxation for 0.5 min. The total duration of the microwave heating was 6 min. Then the reactor was cooled to room temperature. The fluffy layer of silver nanowires formed on the surface was separated, washed with deionized water and acetone, dried and redispersed in isopropyl alcohol.

### 2.3. Obtaining of Optically Transparent Electroconductive Films on the Surface of a Polyethyleneterephthalate Substrate

To obtain functional films of titanium dioxide, a sol with the known titanium concentration was deposited on the substrate surface and evenly distributed on the surface with an air brush. Then, the obtained film was dried in a drying box in an air environment at the temperature of 70 °C. To obtain a layer of silver wires, an aliquot of silver nanowire suspension with the known weight concentration was deposited on the substrate surface and evenly distributed over the surface also with an air brush. The concentration was controlled by visually monitoring when percolation was achieved.

### 2.4. Experimental Apparatuses and Methods

The film transmission coefficients in UV, visible and near IR-region of the spectrum were studied with spectrophotometers Cary 100 and ЛOMOБИK (LOMO BIK). The studies of dielectric characteristics of the films at room temperature were conducted with an RCL meter E7-20. For dielectric measurements, we used a cell with cylindrical stainless-steel electrodes. The electrodes were forced against each other with a strong spring. In this case, we applied a polyethylene terephthalate film (with an indium-tin oxide layer deposited on it by magnetron deposition) as the platform for composite coating formation. This conductive layer was used as the bottom electrode of the capacitor, which was connected with the sensing wire of the RCL-meter E7-20. The surface of the indium-tin oxide film was first coated with a layer of titanium dioxide by dipping from solution into a layer of silver by the technique described above. The obtained sandwich was fixed in the capacitor cell, and measurements were made.

## 3. Results and Discussion

Optical conductivity is one of the most powerful instruments for studying electronic states in materials. The frequency dependence of dielectric properties reflects the fact that material polarization does not immediately react to the applied electric field. For this reason, the dielectric constant is often viewed as a complex function of the frequency of the applied field. An ideal dielectric is a nonconductive material. Materials with low dielectric losses prevent the spread of electromagnetic energy that could increase their conductivity. The real component of optical conductivity (σ) of a crystal is the function of the image component of dielectric permittivity [26,27]: σ_op_ = Im (ε).

The ratio of direct current conductivity to optical conductivity σ_dc_/σ_op_ is often used as a quality indicator for evaluating properties of transparent electrodes. The high value of σ_dc_/σ_op_ indicates that an increase in the transmission coefficient T is accompanied by low resistance of the conducting layer R_sh_. The interrelation between T, R_sh_ and σ_dc_/σ_op_ in works [26,27] is expressed by the following empirical formula:

$$T={\left(1+\frac{Z_{0}\sigma_{op}}{2R_{sh}\sigma_{dc}}\right)}^{-2}$$

*Z*_0_ = 377 Ohm is the vacuum impedance. However, no direct interrelation has been established between the transmission coefficient and the material electric properties. That is why in practice, the conductivity and transmission coefficient of the films under study are compared with those of indium-tin oxide films. It should be said that for anisotropic systems, such as composites of silver nanowires, carbon nanotubes and graphenes, optical transmission and electric conductivity depend on the measurement direction. Common practice is to measure the surface conductivity of such composites in the XY plane and the axial light transmission along the Z axis, which is not quite correct. That is why in this work, we used optical and electric characteristics of films obtained by transmitting light and electric current along the Z axis.

**Figure 5:**
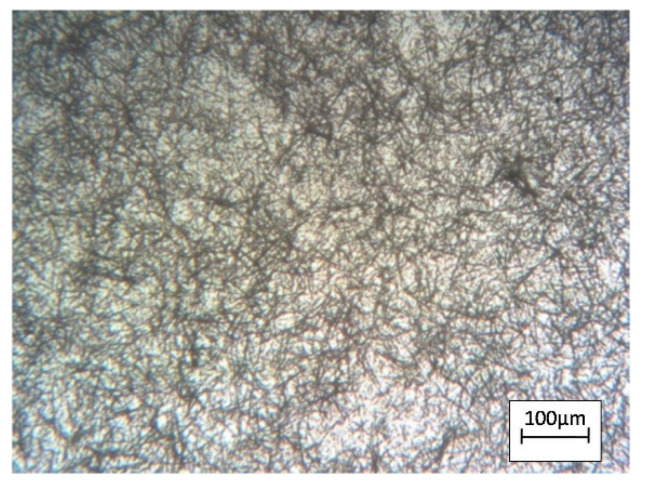
Silver nanowires obtained by adding K_4_Fe(CN)_6_ in the field of an optical microscope.

Figure 5 shows an image of silver nanowires obtained by adding K_4_Fe(CN)_6_ in the field of an optical microscope. As the data shows, the images have no obvious nanowire agglomerates.

**Figure 6:**
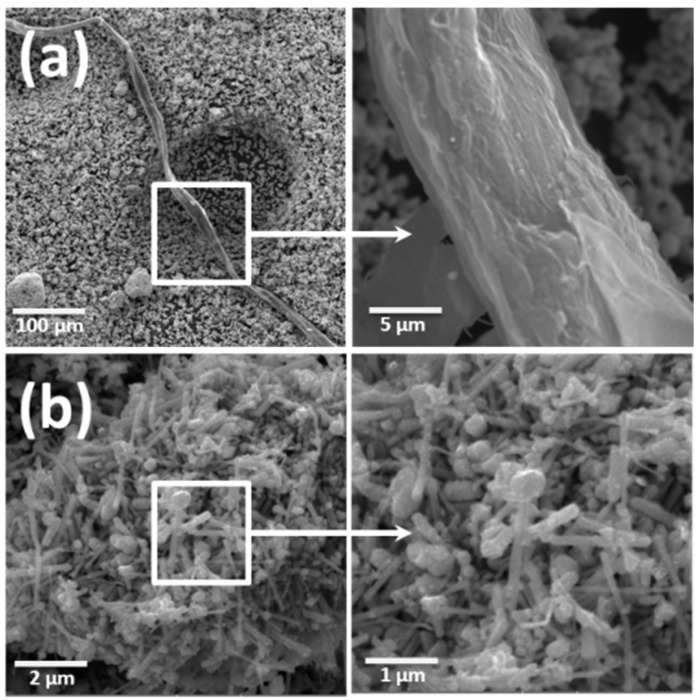
SEM images of silver nanowires: (**a**) Ag_NW; (**b**) Ag_NW_MW.

The structural characteristics of the silver nanowires synthesized by both conventional and microwave methods were studied by scanning electron microscopy (SEM). Figure 6 shows the SEM images of Ag_NW nanowires obtained by the conventional method (Figure 6a) and those obtained by microwave radiation (Figure 6b).

**Figure 7:**
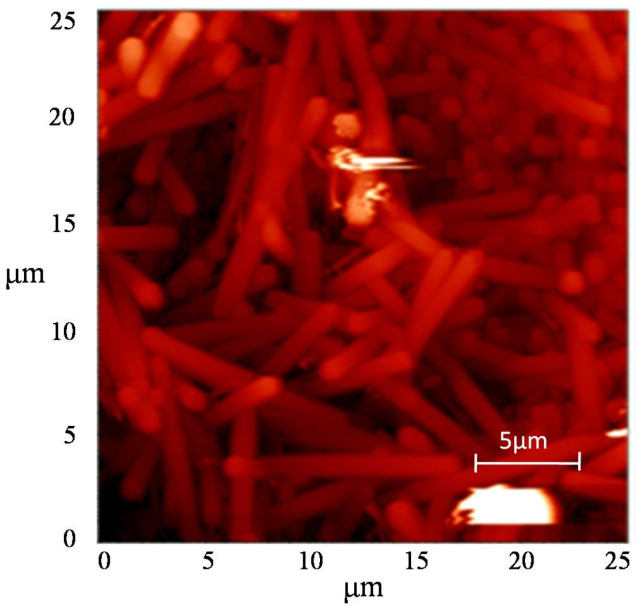
AFM-image of morphology of silver nanowires obtained by adding K_4_Fe(CN)_6_.

According to the atomic-force microscopy data (Figure 7), a silver nanowire has a uniform distribution by thickness of about 200 nm and by length of about 7 μm.

The data of the energy dispersion analysis (Table 1) indicate that the element concentrations during the synthesis procedure and in the obtained material are the same.

**Figure 8:**
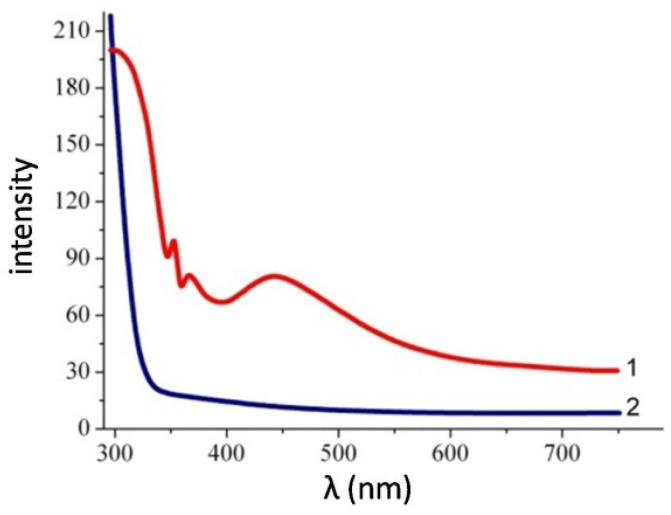
Absorption spectra of films containing 100 mg/m^2^ silver nanoparticles of different morphologies on the TiO_2_ surface: (**1**) TiO_2_/Ag/TiO_2_ nanowires; (**2**) TiO_2_.

**Figure 9:**
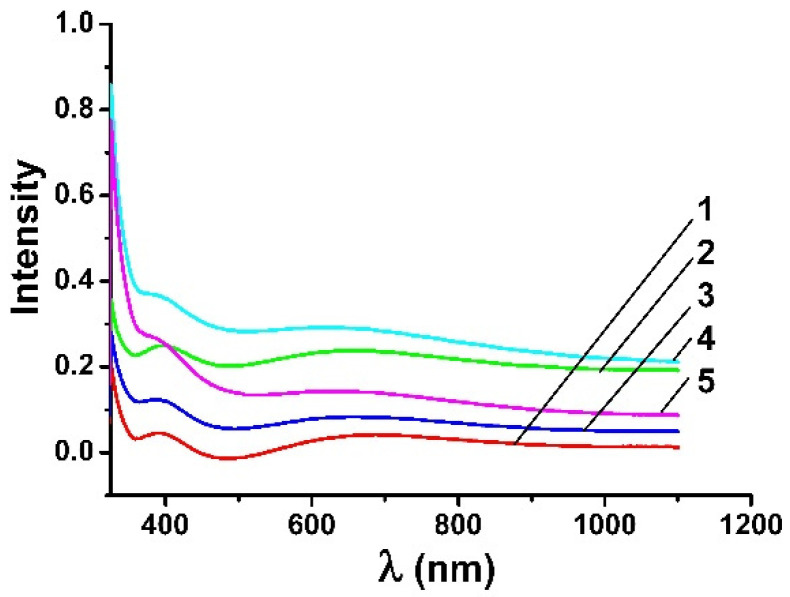
Absorption spectra of films containing: (**1**) TiO_2_ layer; (**2**) two layers: TiO_2_/Ag_NW; (**3**) two layers: TiO_2_/Ag_NW_MW; (**4**) three layers: TiO_2_/Ag_NW/TiO_2_; (**5**) three layers: TiO_2_/Ag_NW_MW/TiO_2_.

As Figure 8 and Figure 9 show, the appearance of absorption maxima on the absorption spectra of the TiO_2_/Ag/TiO_2_ nanocomposite for the film on the polyethylene terephthalate surface is characteristic of elongated anisotropic particles of nanowires. The obtained results confirm the shape of metal nanoparticles obtained by processing the SEM image data. It should be mentioned that the essential factor for achieving Plasmon resonance in nanosystems is obtaining nanoparticles in the range from 10 to 20 nm. All this means that the appearance of characteristic absorption peaks for a composite with silver nanowires can be caused by the surface plasmon effect characterized by quanta of high-density charge vibrations propagating along the boundary of the metal particles’ surface.

**Figure 10:**
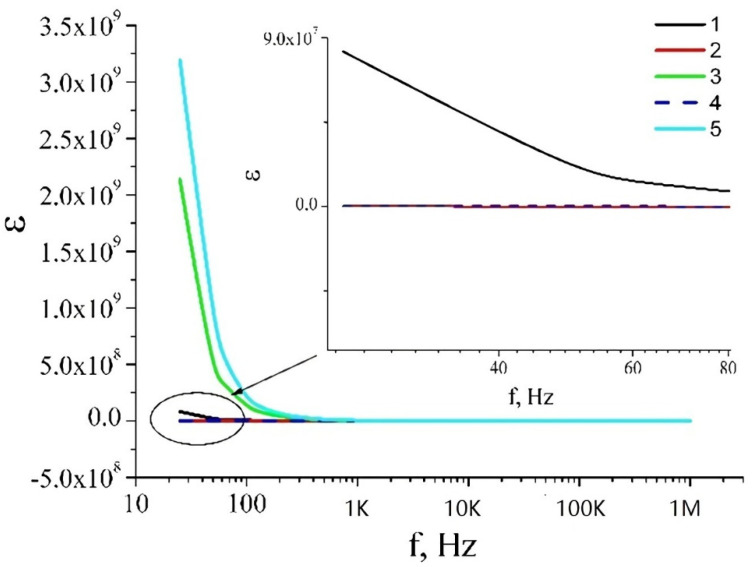
Dependences of dielectric permittivity of films: (**1**) TiO_2_ layer; (**2**) two layers: TiO_2_/Ag_NW; (**3**) two layers: TiO_2_/Ag_NW_MW; (**4**) three layers: TiO_2_/Ag_NW/TiO_2_; (**5**) three layers: TiO_2_/Ag_NW_MW/TiO_2_.

**Figure 11:**
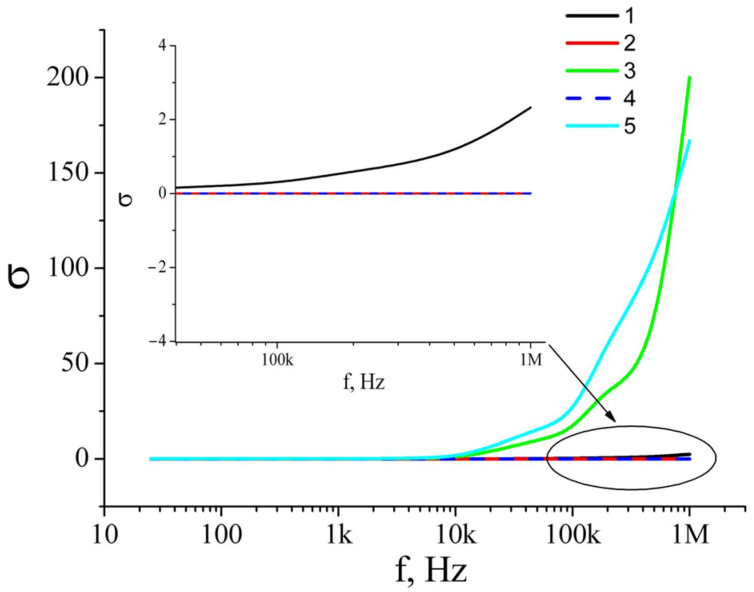
Dependences of films conductivity on the frequency of applied voltage: (**1**) TiO_2_ layer; (**2**) two layers: TiO_2_/Ag_NW; (**3**) two layers: TiO_2_/Ag_NW_MW; (**4**) three layers: TiO_2_/Ag_NW/TiO_2_; (**5**) three layers: TiO_2_/Ag_NW_MW/TiO_2_.

**Figure 12:**
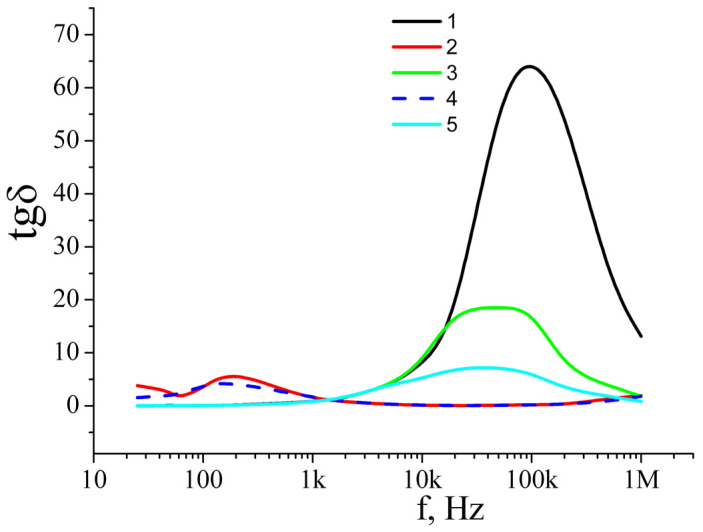
Dependences of dissipation factor of the studied films on frequency: (**1**) TiO_2_ layer; (**2**) two layers: TiO_2_/Ag_NW; (**3**) two layers: TiO_2_/Ag_NW_MW; (**4**) three layers: TiO_2_/Ag_NW/TiO_2_; (**5**) three layers: TiO_2_/Ag_NW_MW/TiO_2_.

The frequency-dependent conductivity and dielectric permittivity of a film are, to a large extent, determined by the Maxwell–Wagner–Sillars mechanism [28,29,30] (Figure 10, Figure 11 and Figure 12). A big difference in the conductivity between a silver nanowire and titanium dioxide leads to large charge accumulation at the interface because it prevents the free current flow across the interface. The accumulated charges are directly proportional to the difference in conductivities between the metal and dielectric phases, and the interface behaves as a nanocapacitor. The formation of numerous nanocapacitors at the interface of silver nanowires and titanium dioxide is realized through the Maxwell–Wagner–Sillars mechanism. For illustrative purposes, we can use an RC scheme to present the interface and its behavior when alternating current flows through it. In this case, the nanocapacitor can be considered a series connection of a plane capacitor (C) and a resistor (R). The plane capacitor denotes the function of accumulation of electric charges, while the resistor denotes the function of resistance to the current. When alternating current flows through such scheme, at a certain frequency the electrons jump over the nanocapacitor, and the conductivity of the alternating current increases, but the accumulated charges decrease. As a result, this leads to higher conductivity in the interlayer and reduces the dielectric permittivity with a frequency increase.

Films of TiO_2_/Ag and TiO_2_/Ag/TiO_2_ nanoheterostructures have been obtained on a flexible polyethylene terephthalate substrate and their dielectric and optical properties were studied. We also studied the effect of the layer-by-layer introduction of silver nanoparticles of anisotropic structure and those in the form of nanowires into the structure of TiO_2_/Ag and TiO_2_/Ag/TiO_2_ nanoheterostructure films on the optical and dielectric properties of materials.

## 4. Conclusions

We studied a multilayer TiO_2_/Ag/TiO_2_ heterostructure with a silver nanowire filler as the transparent composite electrode that demonstrated high conductivity and good transparency. A layer of silver nanowires between the TiO_2_ layers sharply reduces the composite resistance without a significant decrease in the transparency. The TiO_2_ film uniformly coats the silver nanowires filling the cavities and covering the transitions between the Ag nanoparticles. The upper TiO_2_ layer that fills the cavities between the silver nanowires facilitates concentration of charge carriers on the interphase boundary. The resulting interfacial polarization on the interphase boundary metal semiconductor, in accordance with the Maxwell–Wagner–Sillars theory, with an increase in the frequency of the alternating current flowing in the system, leads to conductivity growth and reductions in dielectric permittivity.

## Figures and Tables

**Table 1:** Properties of the sintered specimens.

Element, wt (%)	Ag_NW	Ag_NW_MW
Ag	93.38	98.16
O	6.62	1.84
Sum	100	100

## Data Availability

The data presented in this study are available in the article.

## References

[1] Jung S., Lee S., Song M., Kim D.G., You D.S., Kim J.K., Kang J.W. (2014). Extremely flexible transparent conducting electrodes for organic devices. Adv. Energy Mater..

[2] Krantz J., Stubhan T., Richter M., Spallek S., Litzov I., Matt G.J., Brabec C.J. (2013). Spray-coated silver nanowires as top electrode layer in semitransparent P3HT: PCBM-based organic solar cell devices. Adv. Funct. Mater..

[3] Nam S., Song M., Kim D.H., Cho B., Lee H.M., Kwon J.D., Kim C.S. (2014). Ultrasmooth, extremely deformable and shape recoverable Ag nanowire embedded transparent electrode. Sci. Rep..

[4] Chang J.H., Chiang K.M., Kang H.W., Chi W.J., Chang J.H., Wu C.I., Lin H.W. (2015). A solution-processed molybdenum oxide treated silver nanowire network: A highly conductive transparent conducting electrode with superior mechanical and hole injection properties. Nanoscale.

[5] Shim B.S., Zhu J., Jan E., Critchley K., Kotov N.A. (2010). Transparent conductors from layer-by-layer assembled SWNT films: Importance of mechanical properties and a new figure of merit. ACS Nano.

[6] Patel K., Tyagi P.K. (2015). Multilayer graphene as a transparent conducting electrode in silicon heterojunction solar cells. AIP Adv..

[7] Kang M.G., Kim M.S., Kim J., Guo L.J. (2008). Organic solar cells using nanoimprinted transparent metal electrodes. Adv. Mater..

[8] Lipomi D.J., Vosgueritchian M., Tee B.C., Hellstrom S.L., Lee J.A., Fox C.H., Bao Z. (2011). Skin-like pressure and strain sensors based on transparent elastic films of carbon nanotubes. Nat. Nanotechnol..

[9] Kumar A., Zhou C. (2010). The race to replace tin-doped indium oxide: Which material will win?. ACS Nano.

[10] Guo C.F., Ren Z. (2015). Flexible transparent conductors based on metal nanowire networks. Mater. Today.

[11] Davydova O.I., Kraev A.S., Evdokimova O.L., Gerasimova T.V., Agafonov A.V. (2016). Solution method for production of optically active multilayer titanium dioxide -nanosilver coatings onto polyether substrate. Izv. Vyssh. Uchebn. Zaved. Khim. Khim. Tekhnol..

[12] Davydova O.I., Agafonov A.V. (2016). Growth of optically active multilayer metal oxide films on a plastic substrate. Inorg. Mater..

[13] Sun Y., Yin Y., Mayers B.T., Herricks T., Xia Y. (2002). Uniform silver nanowires synthesis by reducing AgNO3 with ethylene glycol in the presence of seeds and poly (vinyl pyrrolidone). Chem. Mater..

[14] Sun Y., Xia Y. (2002). Large-scale synthesis of uniform silver nanowires through a soft, self-seeding, polyol process. Adv. Mater..

[15] Zhu J.J., Kan C.X., Wan J.G., Han M., Wang G.H. (2011). High-yield synthesis of uniform Ag nanowires with high aspect ratios by introducing the long-chain PVP in an improved polyol process. J. Nanomater..

[16] Liu C.H., Yu X. (2011). Silver nanowire-based transparent, flexible, and conductive thin film. Nanoscale Res. Lett..

[17] Chen C., Wang L., Jiang G., Yang Q., Wang J., Yu H., Chen X. (2005). The influence of seeding conditions and shielding gas atmosphere on the synthesis of silver nanowires through the polyol process. Nanotechnology.

[18] Wiley B., Herricks T., Sun Y., Xia Y. (2004). Polyol synthesis of silver nanoparticles: Use of chloride and oxygen to promote the formation of single-crystal, truncated cubes and tetrahedrons. Nano Lett..

[19] Wiley B., Sun Y., Xia Y. (2005). Polyol synthesis of silver nanostructures: Control of product morphology with Fe (II) or Fe (III) species. Langmuir.

[20] Sun Y., Mayers B., Herricks T., Xia Y. (2003). Polyol synthesis of uniform silver nanowires: A plausible growth mechanism and the supporting evidence. Nano Lett..

[21] Wiley B., Sun Y., Mayers B., Xia Y. (2005). Shape-controlled synthesis of metal nanostructures: The case of silver. Chem. Eur. J..

[22] Sau T.K., Murphy C.J. (2004). Room temperature, high-yield synthesis of multiple shapes of gold nanoparticles in aqueous solution. J. Am. Chem. Soc..

[23] Pileni M.P. (2003). The role of soft colloidal templates in controlling the size and shape of inorganic nanocrystals. Nat. Mater..

[24] Im S.H., Lee Y.T., Wiley B., Xia Y. (2005). Large-scale synthesis of silver nanocubes: The role of HCl in promoting cube perfection and monodispersity. Angew. Chem..

[25] Galkina O.L., Vinogradov V.V., Vinogradov A.V., Agafonov A.V. (2012). Development of the low-temperature sol-gel synthesis of TiO_2_ to provide self-cleaning effect on the textile materials. Nanotechnol. Russ..

[26] Hu L., Hecht D.S., Grüner G. (2004). Percolation in transparent and conducting carbon nanotube networks. Nano Lett..

[27] Lyons P.E., De S., Elias J., Schamel M., Philippe L., Bellew A.T., Coleman J.N. (2011). High-performance transparent conductors from networks of gold nanowires. J. Phys. Chem. Lett..

[28] Maxwell J.C. (1891). A treatise on Electricity and Magnetism. Art.

[29] Sillers R.W. (1936). The properties of a dielectric containing semiconducting particles of various shapes. J. Inst. Electr. Eng..

[30] Caswell K.K., Bender C.M., Murphy C.J. (2003). Seedless, surfactantless wet chemical synthesis of silver nanowires. Nano Lett..

